# Всем ли нужен витамин D? Высокая распространенность дефицита CYP24A1 в российской популяции

**DOI:** 10.14341/probl13561

**Published:** 2025-12-02

**Authors:** К. С. Куликова, С. В. Папиж, А. В. Поляков, А. В. Марахонов, Н. С. Бескоровайный, М. В. Шумихина, Л. С. Созаева, Е. Б. Фролова, Н. Ю. Калинченко, Э. А. Янар, Л. Я. Рожинская, Ю. В. Тихонович, В. П. Богданов, В. А. Иоутси, А. Н. Тюльпаков

**Affiliations:** Медико-генетический научный центр им. акад. Н.П. Бочкова; Национальный медицинский исследовательский центр эндокринологии им. акад. И.И. Дедова; Российская детская клиническая больницаРоссия; Centre for Medical Genetics; Endocrinology Research Centre; Russian Children’s Clinical Hospital - Pirogov Russian National Research Medical UniversityRussian Federation; Научно-исследовательский клинический институт педиатрии им. акад. Ю.Е. ВельтищеваРоссия; Veltischev Research Clinical Institute for Pediatrics and Pediatric SurgeryRussian Federation; Медико-генетический научный центр им. акад. Н.П. БочковаРоссия; Centre for Medical GeneticsRussian Federation; Детская городская клиническая больница им. Н.Ф. ФилатоваРоссия; N.F. Filatov Children City HospitalRussian Federation; Национальный медицинский исследовательский центр эндокринологии им. акад. И.И. ДедоваРоссия; Endocrinology Research CentreRussian Federation; Национальный медицинский исследовательский центр здоровья детейРоссия; National Medical Research Center of Children HealthRussian Federation; Первый Московский государственный медицинский университет им. И.М. Сеченова (Сеченовский Университет)Россия; I.M. Sechenov First Moscow State Medical University (Sechenov University)Russian Federation; Московский физико-технический институт; Федеральный исследовательский центр оригинальных и перспективных биомедицинских и фармацевтических технологийРоссия; Moscow Institute of Physics and Technology; Federal Research Center for Innovator and Emerging Biomedical and Pharmaceutical TechnologiesRussian Federation; Медико-генетический научный центр им. акад. Н.П. Бочкова; Российская детская клиническая больницаРоссия; Centre for Medical Genetics; Russian Children’s Clinical Hospital - Pirogov Russian National Research Medical UniversityRussian Federation

**Keywords:** CYP24A1, дефицит 24-гидроксилазы, инфантильная гиперкальциемия 1 типа, гиперкальциурия, частота, нефрокальциноз, нефролитиаз, ПТГ-независимая гиперкальциемия, витамин D, холекальциферол, интоксикация, 25(ОН)D3:24, 25(OH)2D3, CYP24A1, 24-hydroxylase deficiency, infantile hypercalcemia type 1, hypercalciuria, frequency, nephrocalcinosis, nephrolithiasis, PTH-independent hypercalcemia, vitamin D, cholecalcipherol, intoxication, 25(ОН)D3:24, 25(OH)2D3

## Abstract

Дефицит 24-гидроксилазы (инфантильная гиперкальциемия тип 1) — это наследственное заболевание, ассоциированное с дефектами в гене CYP24A1, приводящими к нарушению инактивации метаболитов витамина D с развитием гиперкальциемии, нефрокальциноза и нефролитиаза. По анализу историй болезни 44 пациентов (27 мужского и 17 женского пола, возраст: 3 месяца–32 года) наибольшую группу (n=41) составили пациенты детского возраста. 16 детей в возрасте до 1 года демонстрировали наиболее яркие клинические проявления заболевания. Основные жалобы на момент обследования: снижение аппетита, задержка физического и психомоторного развития, признаки острого пиелонефрита или персистирующей абактериальной асимптоматической лейкоцитурии и/или нефрокальциноза (у взрослых — нефролитиаза). Холекальциферол в профилактической дозе на момент дебюта заболевания принимали 70,5% детей, Ме длительности приема — 6 [3; 9] месяцев. Гиперкальциемия зафиксирована у 88,6%, Ме Са общего 2,9 [2,65; 4,03] ммоль/л. Ме соотношения 25(OH)D3:24,25(OH)2D3 — 340,65 [132,2; 630,75] (n=10). Гиперкальциурия выявлена в 45,5%, нефрокальциноз или нефролитиаза — в 95% случаев.

Частыми мутациями в гене CYP24A1 оказались p.Arg396Trp (66%) и p.Glu143del. (27%), расчетная частота дефицита 24-гидроксилазы в российской популяции на их основе составила 1 на 10 900 новорожденных, гетерозиготного носительства — 1 на 53 человека.

Впервые проведен расчет частоты дефицита 24-гидроксилазы в российской популяции. Учитывая высокую распространенность заболевания и гетерозиготного носительства, мы предлагаем проводить молекулярно-генетическое тестирование на наличие патогенных вариантов p.Arg396Trp и p.Glu143del в гене CYP24A1 у новорожденных.

## Abstract (en)

24-hydroxylase deficiency (or infantile hypercalcemia type 1) is an inherited disease associated with biallelic loss-of-function CYP24A1 mutations lead to impaired inactivation of vitamin D metabolites and characterised hypercalcemia, nephrocalcinosis and/or urolithiasis. We present 44 patients, the largest group (n=41) consisted of children. The main complaints at the time of examination was: weight loss, refusal to eat, delayed physical and/or psychomotor development, signs of urinary tract infection and/or nephrocalcinosis (in adults — urolithiasis). Hypercalcemia was detected in 88.6%, with Me 2.9 [2.65; 4.03] mmol/L. Me of the 25(OH)D3:24.25(OH)2D3 ratio was 340.65 [132.2; 630.75] (n=10). Hypercalciuria was detected in 59%, nephrocalcinosis or urolithiasis in 95% of cases. Frequent mutations in the CYP24A1 gene were p.Arg396Trp (66%) and p.Glu143del. (27%). Incidence of 24-hydroxylase deficiency in Russian population was 1:10900 estimated on the basis of these alleles of CYP24A1, overall carrier frequency for these mutations in CYP24A1 was 1 in 53 people. In conclusion, we propose to conduct molecular testing for the presence of pathogenic variants p.Arg396Trp and p.Glu143del in CYP24A1 during neonatal screening, due to the high expected frequency of 24-hydroxylase deficiency and heterozygous carriage of pathogenic variants of CYP24A1 in Russia.

## АКТУАЛЬНОСТЬ

Метаболизм витамина D представляет собой гормонально регулируемую систему, связывающую обмен кальция и фосфата. К нарушениям метаболизма витамина D относятся дефекты активации и действия витамина D, проявляющиеся в виде различных форм витамин D-зависимого рахита, а также расстройства с повышенной активацией витамина D. К приобретенным и наследственным состояниям, приводящим к гипервитаминозу D, относятся интоксикация витамином D, гранулематозные заболевания и идиопатическая инфантильная гиперкальциемия, которая может быть вызвана либо дефектом элиминации витамина D, либо первичным дефектом реабсорбции фосфата в почках [1]. Инактивация витамина D осуществляется посредством работы 24-гидроксилазы (CYP24A1). Клинический спектр состояний, вызванных биаллельными мутациями в гене CYP24A1 (MIM 126065), варьирует от идиопатической инфантильной гиперкальциемии 1 типа у детей до возвратного нефролитиаза и/или медуллярного нефрокальциноза с гиперкальциурией во взрослом возрасте [2–4].

Впервые в Великобритании в 1950-х годах обратили внимание на наличие связи между гиперкальциемией у некоторых детей и приемом витамина D для профилактики рахита [5]. С аналогичным «эндемическим» состоянием столкнулись позднее и в других странах. В 2011 г. Schlingmann et al. сообщили о генетической верификации инфантильной гиперкальциемии по результатам обследования 6 пациентов, у которых были обнаружены пять различных патогенных вариантов в гене CYP24A1 [6]. Данная патология первоначально получила название инфантильная гиперкальциемия 1 типа. К настоящему времени в литературе представлено более 64 публикаций с описанием более 200 пациентов с гиперкальциемией в результате врожденного дефицита 24-гидроксилазы [2][3]. Анализ литературных данных показал, что первый пик клинических проявлений приходится на младенчество, а второй — на взрослый возраст около 20 лет [3]. Актуальным и нерешенным остается вопрос о проявлениях заболевания у гетерозиготных носителей мутаций в гене CYP24A1 [7].

По некоторым данным, расчетная частота заболевания оценивается как 1:60 000 новорожденных, однако имеются публикации, где представлена более высокая расчетная частота. Например, в польской популяции заболеваемость, оцененная на основе частоты аллелей патогенного варианта Arg396Trp в гене CYP24A1, составляет 1:32 465 новорожденных, а частота носительства 1:90 соответственно [4]. Исследований по изучению частоты заболевания и оценке частоты носительства патогенных вариантов в гене CYP24A1 в российской популяции ранее не проводилось.

В настоящей статье нами представлен анализ клинических, биохимических и молекулярно-генетических данных у 44 пациентов с дефицитом 24-гидроксилазы. Дополнительно, используя российские базы данных экзомов и геномов, нами проведена оценка частоты гетерозиготного носительства патогенных вариантов в гене CYP24A1 и рассчитана частота заболевания.

## МАТЕРИАЛЫ И МЕТОДЫ

В исследование включено 44 пациента из 43 семей с гиперкальциемией (>2,6 ммоль/л) или с анамнезом острой или хронической гиперкальциемии в сочетании с уровнем ПТГ менее 20 пг/мл, а также признаками нефрокальциноза и/или нефролитиаза. Данные о клинических симптомах и биохимических параметрах (кальций и фосфат в сыворотке и моче, уровень паратгормона, витамина D и др.) собирались ретроспективно с использованием медицинских записей.

Некоторым пациентам (n=10) выполнен профиль метаболитов витамина D в сыворотке крови методом жидкостной хроматографии — тандемной масс-спектрометрии (ЖХ-МС/МС). Результаты выражены как молярное отношение 25(OH)D3:24,25(OH)2D3. Значения менее 25 указывают на отсутствие дефекта в активности 24-гидроксилазы, и считались нормальными [8].

Всем пациентам было проведено генетическое тестирование на наличие мутаций в гене CYP24A1. Молекулярно-генетический анализ проводился после получения письменного информированного согласия пациента и/или его законного представителя. Геномную ДНК выделяли из лейкоцитов периферический крови наборами PureLink® Genomic DNA Mini Kit (Thermo Scientific, Waltham, MA, USA). Генетическое тестирование выполнялось с помощью следующих методик: прямое автоматическое секвенирование гена CYP24A1 по Сэнгеру (n=2), массовое параллельное секвенирование с исследованием мутаций в 22 генах, ассоциированных с нарушениями фосфорно-кальциевого обмена (n=35), или полное секвенирование экзома (n=7). Оценка патогенности варианта нуклеотидной последовательности проводилась согласно международным и российским рекомендациям [9][10]. Нумерация кодирующей последовательности гена CYP24A1 дана по референсу NM_000782.5 (http://www.ncbi.nlm.nih.gov/genbank).

Для оценки частоты гетерозиготного носительства описанных патогенных вариантов в гене CYP24A1 был выполнен поиск данных вариантов в 5704 образцах ДНК (11 408 аллелей) по базе RuExac [11] и 3000 образцах ДНК (6000 аллелей) по базе данных ЦГРМ «ГЕНЕТИКО». Всего для анализа использовано 8704 образца (17 408 аллелей).

## РЕЗУЛЬТАТЫ

Клиническая характеристика пациентов представлена в таблице 1, из которых 26 были ранее описаны [12–14]. Мы зарегистрировали 44 пациента: 27 мужского (61,3%) и 17 женского (38,7%) пола, включая 41 ребенка (93%) в возрасте от 3 месяцев до 11 лет (Ме возраста 20 [ 9; 63] месяцев) и 3 взрослых (7%) от 20 до 32 лет. Многочисленную группу (36% случаев) с наиболее яркими клиническими проявлениями заболевания на фоне тяжелой гиперкальциемии представляли дети до 1 года.

**Table:** Таблица 1. Клинические, биохимико-гормональные особенности пациентов с биаллельными вариантами в гене CYP24A1 Примечание: м — мужской; ж — женский; Са общий сыворотки, ммоль/л: 2,1–2,55; фосфор неорганический сыворотки, ммоль/л (референсные значения даны при измерении на аппарате Abbott ARCHITECTc8000): 15 дней–1 год: 1,54–2,72, 1 — <5 лет: 1,38–2,19, 5 — <13 лет: 1,33–1,92, 13 — <16 лет: 1,02–1,79; 16 — <19 лет: 0,95 — 1,62; 0,8–1,45; ПТГ (паратгормон), пг/мл: 15–65; 25(ОН)D сыворотки, нг/мл: 30–100; 25(OH)D/24,25(OH)2D соотношение 5–25; н/д — нет данных.

Пациент	Пол	Доза холекальциферола, МЕ/сут	Возраст манифестации (месяцы)	Возраст постановки диагноза (месяцы, лет)	Са общ. сыв., ммоль/л	Фосфор неорг. сыв., ммоль/л	ПТГ сыв., пг/мл	25(ОН)D сыв., нг/мл	25(ОН)D:24,25(ОН)2D	Гиперкальциурия (+/-)	Нефрокальциноз	Мутация в гене CYP24A1 (NM_000782.5)
кДНК	Белок
1	м	1000	5	9 мес.	5,2	-	0,8	153	-	+	+	c.[ 107delC]c.[ 1186C>T]	p.[ Pro36Leufs*11]p.[ Arg396Trp]
2	м	-	5	9,4 года	3,69	1,56	9,3	48,2	-	+	+	c.[ 182C>A]c.[ 1186C>T]	p.[ Pro61His]p.[ Arg396Trp]
3	ж	1000	77	7,3 года	2,42	1,59	<3	55	-	-	+	c.[ 1156A>T]c.[ 1286T>C]	p.[ Arg386Trp]p.[ Phe429Ser]
4	м	500	10	4,4 года	-	1,85	10	-	-	-	+	c.[ 1186C>T]c.[ 1186C>T]	p.[ Arg396Trp]p.[ Arg396Trp]
5	ж	1000	14	1,3 года	2,96	1,86	13,3	15,75	-	+	+	c.[ 1186C>T]c.[ 1186C>T]	p.[ Arg396Trp]p.[ Arg396Trp]
6	м	1000	10	1,4 года	3,41	2,07	14,5	22,16	-	-	+	c.[ 1186C>T]c.[ 1186C>T]	p.[ Arg396Trp]p.[ Arg396Trp]
7	м	1000	4	11 мес.	5,19	0,95	15,44	>160	649	-	+	c.[ 1186C>T]c.[ 1186C>T]	p.[ Arg396Trp]p.[ Arg396Trp]
8	м	-	-	31 год	2,9	-	1,6		126,3	н/д	+	c.[ 1186C>T]c.[ 1186C>T]	p.[ Arg396Trp]p.[ Arg396Trp]
9	м	1000	6	6 мес.	6,39	-	3	214	-	+	+	c.[ 1186C>T]c.[ 1186C>T]	p.[ Arg396Trp]p.[ Arg396Trp]
10	ж	1500	12	1,2 года	4,34	1,64	12,3	-	-	+	+	c.[ 1186C>T]c.[ 1186C>T]	p.[ Arg396Trp]p.[ Arg396Trp]
11	ж	0	60	7 лет	2,57	1,87	7,93		576	+	+	c.[ 1186C>T]c.[ 1186C>T]	p.[ Arg396Trp]p.[ Arg396Trp]
12	м	500	6	1 год	4,73	-	4,14	>170,4	876	-	+	c.[ 1186C>T]c.[ 1186C>T]	p.[ Arg396Trp]p.[ Arg396Trp]
13	м	1000	3	6 мес.	5,15	1,57	<3	144	898	+	+	c.[ 1186C>T]c.[ 1186C>T]	p.[ Arg396Trp]p.[ Arg396Trp]
14	м	-	-	20 лет	2,69	1,13	4,41	53,1	467	+	+	c.[ 1186C>T]c.[ 1186C>T]	p.[ Arg396Trp]p.[ Arg396Trp]
15	м	1000	108	9 лет	2,62	1,97	10,66	41,06	-	-	+	c.[ 1186C>T]c.[ 1186C>T]	p.[ Arg396Trp]p.[ Arg396Trp]
16	м	-	-	2 года	2,65	1,89	7,09	-	-	н/д	+	c.[ 1186C>T]c.[ 1226T>C]	p.[ Arg396Trp]p.[ Leu409Ser]
17	м	500	3	8 мес.	5,3	2,9	<3	160	-	н/д	+	c.[ 1186C>T]c.[ 1226T>C]	p.[ Arg396Trp]p.[ Leu409Ser]
18	ж	-	3	3 года	4.52	1,39	1,88	190	-	-	+	c.[ 1186C>T]c.[ 1226T>C]	p.[ Arg396Trp]p.[ Leu409Ser]
19	м	1000	4	5 лет	2,71	2,26	18	22	-	+	+	с.[ 1186C>T]; [ 1226T>C]	p.[ Arg396Trp]p.[ Leu409Ser]
20	м	-	-	20 лет	2,9	-	4,3		95,6	+	+	c.[ 1186C>T]c.[ 1315C>T]	p.[ Arg396Trp]p.[ Arg439Cys]
21	ж	-	62	5,3 года	2,66	1,36	15	-	-	+	+	c.[ 1186C>T]c.[ 233G>T]	p.[ Arg396Trp]p.[ Gly78Val]
22	ж	2500	56	5,8 года	2,61	1,7	-	-	-	+	+	c.[ 1186C>T]c.[ 428_430delAAG]	p.[ Arg396Trp]p.[ Glu143del]
23	ж	-	10	1 год	2,9	-	8	22,1	-	-	+	c.[ 1186C>T]c.[ 428_430delAAG]	p.[ Arg396Trp]p.[ Glu143del]
24	м	1000	60	5 лет	2,33	1,79		40,01	-	-	+	c.[ 1186C>T]c.[ 428_430delAAG]	p.[ Arg396Trp]p.[ Glu143del]
25	м	2000	6	2,5 года	2,76	1,7	7	32,6	-	+	+	c.[ 1186C>T]c.[ 428_430delAAG]	p.[ Arg396Trp]p.[ Glu143del]
26	ж	2000	3	6 мес.	4,7	1,58	-	171,4	-	+	+	с.[ 1186C>T]c.[ 443T>C]	p.[ Arg396Trp]p.[ Leu148Pro]
27	ж	1000	4	2 года	2,62	1,92	6,2	37	-	+	+	c.[ 1187G>A]c.[ 1508C>T]	p.[ Arg396Gln]p.[ Pro503Leu]
28	ж	1500	4	1,9 года	2,74	1,24	5.37	46,9	-	+	+	c.[ 1187G>A]c.[ 1524delC]	p.[ Arg396Gln]p.[ Term515Next*?]
29	м	1000	36	3 год 10 мес.	3,5	1,59	<3	68	-	+	+	c.[ 1226T>C]c.[ 1379G>T]	p.[ Leu409Ser]p.[ Arg460Ile]
30	м	1500	9	3,3 года	3,74	-	3	-	-	+	+	c.[ 1396C>T]c.[ 1396C>T]	p.[ Arg466*]p.[ Arg466*]
31	ж	1000	24	8 лет11 мес.	2,55	1,74	10,3	19,4	-	+	+	c.[ 296Т>C]c.[ 1186C>T]	p.[ Met99Thr]p.[ Arg396Trp]
32	ж	5000	6	7,6 года	2,85	1,99	13,1	19,4	-	н/д	+	c.[ 296Т>C]c.[ 428_430delAAG]	p.[ Met99Thr]p.[ Glu143del]
33	м	-	6	2,5 года	5,2	-	7,8	26,3		-	+	c.[ 400T>G]c.[ 428_430delAAG]	p.[ Trp134Gly]p.[ Glu143del]
34	м	1000	5	1,3 года	3,08	1,75	<3	-	-	+	+	c.[ 428_430delAAG]c.[ 428_430delAAG]	p.[ Glu143del]p.[ Glu143del]
35	ж	-	84	12,3 года	2,59	1,48	14,2	15	-	+	+	c.[ 428_430delAAG]c.[ 428_430delAAG]	p.[ Glu143del]p.[ Glu143del]
36	м	1250	10	1,2 года	4,03		2,2	67,35	-	+	+	c.[ 428_430delAAG]c.[ 428_430delAAG]	p.[ Glu143del]p.[ Glu143del]
37	м	1000	4	9 мес.	4,14		3	113	214,3	н/д	-	c.[ 428_430delAAG]c.[ 428_430delAAG]	p.[ Glu143del]p.[ Glu143del]
38	ж	-	62	11 лет	2,65	1,78		27,3	-	+	+	c.[ 428_430delAAG]c.[ 428_430delAAG]	p.[ Glu143del]p.[ Glu143del]
39	ж	-	108	10 лет	2,44	1,74	11,9	-	-	+	+	c.[ 428_430delAAG]c.[ 1315C>T]	p.[ Glu143del]p.[ Arg439Cys]
40	ж	1000	1	4 мес.	3,04	2,22	3	8,1	27	+	+	c.[ 443T>C]c.[ 1410dupT]	p.[ Leu148Pro]c.[ Gln471Serfs*21]
41	м	2000	12	9 лет	2,93	2,22	5,9	21,05	-	+	+	c.[ 443T>C]c.[ 475C>T]	p.[ Leu148Pro]c.[ Arg159Trp]
42.1	м	-	72	6 лет	2,43	1,72	12,8	-	-	-	+	c.[ 476G>A]c.[ 1186C>T]	p.[ Arg159Gln]c.[ Arg396Trp]
42.2	м	-	-	4 года	-	1,25	12,4	-	-	-	-	c.[ 476G>A]c.[ 1186C>T]	p.[ Arg159Gln]c.[ Arg396Trp]
44	м	500	6,5	1,9 года	3,31	-	2,4	-	150	-	+	c.[ 964G>A]c.[ 1186C>T]	p.[ Glu322Lys]c.[ Arg396Trp]

Основными причинами, послужившими поводом для инициации обследования детей, были следующие: снижение аппетита (n=12), недостаточная прибавка в массе тела (n=14), задержка физического и/или психомоторного развития у детей (n=12), признаки острого пиелонефрита или персистирующей абактериальной асимптоматической лейкоцитурии (n=12) и наличие признаков нефрокальциноза и/или улитиаза по данным УЗИ почек (n=42). Взрослые пациенты были обследованы с целью диагностики причин улитиаза.

Медиана возраста манифестации заболевания среди детей составила 9 [ 4,5; 46] месяцев, Ме постановки диагноза с генетической верификацией — 31 [ 12; 72] месяц. Таким образом, в среднем запаздывание в постановке диагноза от момента появления первых симптомов составило 22 [ 4,75; 33] месяца.

Семейный анамнез по улитиазу был отягощен у 10 пациентов. Двое пациентов с мутацией в гене CYP24A1 были родные братья (N42.1, N 42.2), один из которых (N 42.2) без клинических проявлений обследован по причине наличия заболевания у сибса.

Информация о приеме холекальциферола и дозах была доступна в 70,5% случаев (n=31), Ме дозы составила 1000 [ 1000; 1375] МЕ/сут, Ме длительности приема до момента обследования — 6 [ 3; 9] месяцев.

На момент диагностики гиперкальциемия была зафиксирована у 39 пациентов (88,6%), Ме уровня Са общего составила 2,9 [ 2,65; 4,03] ммоль/л, Ca ионизированного — 1,42 [ 1,32; 1,9] ммоль/л. Тяжелая гиперкальциемия (Са общий сыворотки >3,5 ммоль/л) была диагностирована у 13 пациентов (29,5%) в возрасте до 12 месяцев с максимальным уровнем кальция в крови 6,39 ммоль/л у мальчика 6 месяцев (№9), которому по тяжести состояния потребовалась госпитализация в отделение интенсивной терапии. Умеренная гипофосфатемия была у 10 пациентов детского возраста (22,7%), Ме фосфора сыворотки 1,24 [ 1,1; 1,3] ммоль/л. У подавляющего большинства обследованных (n=39) уровень паратгормона в крови был снижен, с Ме ПТГ в крови — 7,86 [ 3,28; 12,4] пг/мл. Значения 25(ОН)D в крови более 150 нг/мл были зафиксированы у 7 детей (15,9%), максимальный уровень 25(ОН)D был 214 нг/мл у ребенка 6 месяцев с тяжелой гиперкальциемией (№9, табл. 1), который к моменту обследования получал холекальциферол в дозе, не превышающей 1000 МЕ/сутки в течение 3 месяцев.

По результатам определения метаболитов витамина D в сыворотке крови у 10 пациентов Ме соотношения 25(OH)D3:24,25(OH)2D3 составила 340,65 [ 132,2; 630,7], у одной пациентки данный показатель был 27 на фоне выраженного дефицита витамина D (№40, табл. 1).

Гиперкальциурия была выявлена у 26 пациентов (59%) по результатам определения кальцийкреатининового соотношения разовой порции мочи и экскреции кальция в суточной порции.

У 42 пациентов, по данным УЗИ почек, отмечались признаки нефрокальциноза и/или нефролитиаза различной степени выраженности.

Всем пациентам выполнен поиск мутаций в гене CYP24A1. У 27 пациентов (61% случаев) патогенные варианты обнаружены в компаунд-гетерозиготном состоянии, у 17 (39%) — в гомозиготном. В общей когорте пациентов выявлен 21 вариант нуклеотидной последовательности в гене CYP24A1, в большинстве случаев (95,2%) мутации оценивались как патогенные или вероятно патогенные [9][10]. Наиболее частым был миссенс-вариант p.Arg396Trp (c.1186C>T), который наблюдался у 29 пациентов (66%) (41/88 аллелей; 12 гомозигот и 17 компаунд-гетерозигот); вторым по частоте был вариант p.Glu143del (c.428_430delAAG), обнаруженный у 12 пациентов (27%) (17/88 аллелей; 5 гомозигот и 7 компаунд-гетерозигот); сочетание этих двух вариантов выявлено у четырех больных. Доля варианта p.Arg396Trp составила 47% от всех хромосом больных с выявленной мутацией, а варианта p.Glu143del — 19%. Таким образом, на долю этих двух вариантов в изученной когорте больных пришлось 65,91% (58/88 аллелей) всех мутантных аллелей.

По данным базы RuExac, частоты вариантов p.Arg396Trp и p.Glu143del составили 0,0046 (52 аллеля из 11 408, 95% ДИ: 0,0034–0,0060) и 0,0014 (16 аллелей из 11 408, 95% ДИ: 0,0008–0,0023) соответственно [16]. По данным базы ЦГРМ «ГЕНЕТИКО», частоты данных вариантов составили 0,0050 (30 аллелей из 6000; 95% ДИ: 0,0034–0,0071) и 0,0020 (12 аллелей из 6000; 95% ДИ: 0,0010–0,0035) соответственно. Поскольку полученные по двум базам данные по частотам достоверно не различаются (p-value=0,6858 и 0,3498 для вариантов p.Arg396Trp и p.Glu143del соответственно), они были объединены. Таким образом, по совокупным данным частота варианта p.Arg396Trp составила 0,0047 (95% ДИ: 0,0037–0,0058), варианта p.Glu143del — 0,0016 (95% ДИ: 0,0011–0,0023), а их суммарная частота — 0,0063 (95% ДИ: 0,0052–0,0076). Поскольку на долю этих двух частых вариантов приходится 66% всех мутантных аллелей в гене CYP24A1, то частота всех причинных вариантов в данном гене в РФ равна 0,0063/0,6591=0,0096 (с учетом 95% ДИ: 0,0079–0,0116). Отсюда популяционная частота гетерозиготного носительства мутаций в гене CYP24A1 равна 0,0190 (с учетом 95% ДИ: 0,0156–0,0229) или каждый 53-й человек является носителем. На основе проведенного анализа расчетная частота дефицита 24-гидроксилазы в России составляет 9,19×10−5 (с учетом 95% ДИ: 6,22×10−5–1,33×10−4) или 1 на 10 900 новорожденных (с учетом 95% ДИ: 1 на 7500–16100 новорожденных).

## ОБСУЖДЕНИЕ

Гиперкальциемия, обусловленная инактивирующими мутациями в гене CYP24A1, относится к наследственным ПТГ-независимым формам гиперкальциемии. В настоящее время обсуждается вопрос о корректности названия данного заболевания как инфантильная гиперкальциемия 1 типа, поскольку симптомы заболевания и установление данного диагноза не ограничиваются только младенческим возрастом, но могут встречаться и у взрослых, некоторые авторы предлагают использовать термины «дефицит 24-гидроксилазы» [15] или «гиперчувствительность к витамину D» [4].

Известно, что дефицит 24-гидроксилазы наследуется по аутосомно-рецессивному типу, и наиболее яркая клиническая картина в результате тяжелой гиперкальциемии характерна для пациентов раннего детского возраста. В исследуемой нами когорте пациентов наиболее высокие значения кальция крови отмечались у детей в первый год жизни. Связанное с возрастом изменение фенотипа было описано ранее и, предположительно, это обусловлено прогрессивным развитием резистентности тканей к витамину D со временем и ускоренным клиренсом кальция у детей более старшего возраста за счет повышения фильтрационной функции почек, что притупляет гиперкальциемическую реакцию у взрослых, но не у младенцев [16]. В большинстве случаев триггерным фактором к развитию гиперкальциемии в младенческом возрасте был прием витамина D, при этом дозы витамина D, которые использовались у пациентов, соответствовали профилактическим, которые по современным международным рекомендациям не требуют назначения каких-либо анализов для оценки их адекватности и безопасности перед началом или во время приема [17]. Представленные в нашем исследовании результаты сопоставимы с имеющимися литературными данными и подтверждают высокую распространенность дефицита 24-гироксилазы в популяции. Кроме того, известно, что у гетерозиготных носителей патогенных вариантов в гене CYP24A1 имеется высокий риск развития нефрокальциноза. Закономерен вопрос о безопасности и рациональности всеобщего унифицированного применения профилактической терапии холекальциферолом. Также необходимо отметить, что одной из групп повышенного внимания по развитию серьезных осложнений являются беременные женщины с биаллельными мутациями в гене CYP24A1. На фоне приема добавок витамина D у женщин с дефектами в гене CYP24A1 может развиться тяжелая гиперкальциемия во время беременности, которая может быть причиной преждевременных родов или задержки внутриутробного развития плода, а также высокого риска развития акушерских осложнений, таких как артериальная гипертензия, преэклампсия и гиперкальциемический криз [18]. По данным систематического обзора Cappellani D. et al., из 20 беременностей, осложненных симптоматической гиперкальциемией на фоне дефицита 24-гидроксилазы, в 11 (55%) случаях произошли самопроизвольные роды, в 7 (35%) случаях — индукция родов и в 2 (10%) случаях — самопроизвольный выкидыш [2]. Отмена добавок витамина D оказалась эффективной в предотвращении симптоматической гиперкальциемии при повторных беременностях и, соответственно, эта стратегия должна быть тщательно рассмотрена.

Учитывая относительно высокую распространенность данной патологии, следует ожидать, что дефицит 24-гидроксилазы может сочетаться и усугублять течение заболеваний, которые изначально ассоциированы с гиперкальциемией, например, с первичным гиперпаратиреозом (ПГПТ), что может существенно влиять на тактику ведения больного. В доступной литературе имеется описание 6 пациентов с сочетанием этих двух состояний, у большинства из них паратиреоидэктомия была первоначально выполнена после диагностики ПГПТ, а после операции из-за сохранения гиперкальциемии был установлен диагноз дефицита 24-гидроксилазы [19], что привело к отмене приема витамина D, ограничению потребления кальция, соблюдению расширенного питьевого режима, в некоторых случаях — применению петлевых диуретиков, бисфосфонатов, глюкокортикоидов и др. Данный факт также подтверждает необходимость расширения диапазона дифференциальной диагностики гиперкальциемии с учетом возможного наличия дефицита 24-гидроксилазы.

В доступной литературе имеются единичные сообщения о долгосрочном наблюдении за пациентами с дефицитом 24-гидроксилазы, которым заболевание было диагностировано в раннем возрасте. Janiec A. et al. (2021) приводят результаты наблюдения группы больных в возрасте от 2 до 34 лет, которые демонстрируют, что у 14 из 18 (77%) субъектов СКФ была <90 мл/мин/1,73 м², а у двух пациентов с мутацией в гене CYP24A1 развилась ХПН, и им была проведена трансплантация почки; УЗИ почек выявило нефрокальциноз у 16 из 18 (88%) пациентов с мутациями в генах CYP24A1 или SLC34A1 [20]. Как известно, дефекты в гене SLC34A1 являются причиной инфантильной гиперкальциемии 2 типа. Авторы также обращают внимание на то, что осложнения в виде нефрокальциноза и ХБП развились у большинства, несмотря на попытки уменьшить воздействие повышенной инсоляции, снижения приема витамина D и добавок кальция. В работе Molin А. et al. распространенность нефролитиаза в течение жизни оценивается в 10–15% [16], Nesterova G. et al. представили частоту почечных камней из-за дефицита CYP24A1 в 4–20% случаев [15]. В нашей работе также продемонстрирована высокая частота нефрокальциноза у детей (до 95% случаев) на момент диагностики, у взрослых (3/44) с дефицитом 24-гидроксилазы в 100% случаев имелась МКБ с подросткового возраста, что подтверждает ключевое значение дефицита CYP24A1 в поражении почек. Кроме нефролитиаза во взрослом возрасте у лиц с данной патологией может отмечаться тенденция к умеренной гиперкальциемии, которая может иметь сезонный характер, например, в летний период при повышенном воздействии солнца, или при приеме добавок витамина D [7]. В 2021 г. Hanna C. et al. опубликовали данные о наличии связи между дефицитом CYP24A1 и кистами почек. Так, среди 16 пациентов с патогенными вариантами в гене CYP24A1 медуллярные и/или кортико-медуллярные кисты присутствовали во всех случаях, медиана возраста при первом обнаружении кисты составила 37 лет (диапазон 3–60 лет), у 80% детей была обнаружена как минимум 1 киста размером ≥5 мм [21]. В нашей группе пациентов только у одного мальчика (N 42.2) с бессимптомным течением заболевания по УЗИ было достоверно зафиксировано анэхогенное образование левой почки до 5,1 мм в диаметре.

В нашей работе анализ уровней метаболитов витамина D в образцах сыворотки показал очень низкие концентрации 24,25(ОН)2D3 практически у всех пациентов (9/10). В 90% случаев у пациентов с доказанным дефицитом 24-гидроксилазы соотношение 25(OH)D3 к 24,25(OH)2D3 было >80. Как известно, данный показатель и его значение более 80 рекомендовано использовать в качестве биохимического критерия заболевания, тогда как у здоровых лиц он не превышает 25 [8]. В одном случае соотношение 25(OH)D3:24,25(OH)2D3 незначительно превышало норму, что было связано с выраженным дефицитом витамина D, при этом уровень 24,25(ОН)2D3 был низким. Применение расчета соотношения 25(OH)D3:24,25(OH)2D3 для диагностики гетерозиготных носителей считается проблематичным, так как в исследованиях статистически значимой разницы по данному параметру между гетерозиготными носителями и здоровыми лицами обнаружено не было [7].

Гипофосфатемия среди нашей когорты выявлена в 22,7% случаев и была характерна для пациентов детского возраста, при этом клинических или рентгенологических признаков рахита не отмечалось. В работе Molin A. et al. сниженный уровень фосфора был выявлен в 27-28% случаев среди пациентов с патогенными вариантами в гене CYP24A1 [22]. Нарушение обмена фосфора с развитием гипофосфатемии является одним из основных биохимических маркеров гипофосфатемического рахита с гиперкальциурией в результате мутаций в гене SLC34A3, а также может быть при инфантильной гиперкальциемии 2 типа при дефектах в гене SLC34A1. Предполагается, что избыток 1,25(OH)2D при дефиците 24-гидроксилазы способствует повышению синтеза фактора роста фибробластов 23 (ФРФ23) и, следовательно, приводит к потере фосфата и гипофосфатемии, однако без развития рахита.

В настоящее время в литературе описано более 20 различных патогенных вариантов в гене CYP24A1 [4], а в базе данных мутаций генов человека аннотировано около 60 мутаций (http://www.hgmd.cf.ac.uk/ac/index.php). В обследуемой нами когорте пациентов из 21 варианта в гене CYP24A1 наиболее частыми были p.Arg396Trp (47%), p.Glu143del (19%), что соотносится с результатами исследований в европейской популяции, и их можно считать «горячими точками» в гене CYP24A1 [2]. В популяции Польши патогенный вариант p.Arg396Trp составляет приблизительно 61,1% аллелей гена CYP24A1, а частота данного аллеля была рассчитана как 0,0034 (0,34%; 1:294) [4]. Проведенные ранее функциональные исследования указанных мутаций in vitro продемонстрировали полное отсутствие или значительное снижение ферментативной активности CYP24A1 [6].

Два самых частых патогенных варианта p.Arg396Trp (c.1186C>T) и p.Glu143del (c.428_430delAAG) в гене CYP24A1 встретились в популяционной выборке россиян с частотами 0,0046 и 0,0014. В базе данных Gnomad в выборке европейцев нефинского происхождения для них представлены частоты 0,00092 и 0,00064. Таким образом, частота варианта p.Glu143del в РФ в два с половиной раза выше, чем в Европе, а варианта p.Arg396Trp в пять раз выше. Соответственно, в РФ следует ожидать существенно большую, чем в европейских странах, и частоту дефицита 24-гидроксилазы.

Большинство пациентов с дефицитом CYP24A1, вероятно, остаются нераспознанными, поскольку в литературе описано всего несколько сотен случаев (например, 221 случай в систематическом обзоре Cappellani et al. [2]), а польское исследование предполагает частоту заболевания в популяции 1 на 32 465 рождений [4]. По результатам проведенного нами исследования, расчетная частота дефицита 24-гидроксилазы оценивается как 1 на 10 900 новорожденных, а гетерозиготное носительство патогенных вариантов в гене CYP24A1 как 1 на 53, что значимо выше, чем представлено в работе из Польши.

Дискутабельным остается вопрос о влиянии моноаллельных мутаций в гене CYP24A1 на фенотип человека [23]. По результатам систематического обзора Cappellani D. et al. у 72 носителей моноаллельных вариантов отмечался более мягкий биохимический и клинический фенотип, однако среди этой группы процент пациентов с нефролитиазом составил 19,4%, что выше, чем в общей популяции [2]. В работе Brancatella et al. при сравнении фенотипа гетерозиготных носителей со здоровыми людьми были получены данные о тенденции к более высокой концентрации кальция крови и уровня 25(OH)D без какой-либо разницы в других биохимических параметрах и наличии/отсутствии нефролитиаза [7]. Невозможность сделать однозначные выводы относительно риска развития осложнений для гетерозиготных носителей мутаций в гене CYP24A1 связана с отсутствием когортных исследований по сопоставлению носителей со здоровыми людьми. Однако результаты даже немногочисленных публикаций указывают на необходимость более тщательного наблюдения не только за пациентами с биаллельными вариантами в гене CYP24A1, но и гетерозиготными носителями из-за потенциального риска развития гиперкальциемии и связанных с ней клинических проявлений при воздействии провоцирующих факторов.

Определенные нами расчетная частота дефицита 24-гидроклилазы и частота носительства в России близки к таковым для таких хорошо известных и социально значимых заболеваний, как фенилкетонурия и муковисцидоз. Обе эти нозологии находятся в программе федерального скрининга новорожденных.

## ЗАКЛЮЧЕНИЕ

Полученные нами данные указывают на высокую частоту дефицита 24-гидроксилазы (1 на 10 900) и частоту гетерозиготного носительства патогенных вариантов в гене CYP24A1 (1 на 53 человека) в российской популяции, которая является весьма высокой для моногенного заболевания и соответствует частотам заболеваний, включенных в неонатальный скрининг в РФ. Кроме того, для заболевания существует эффективный метод профилактики — исключение приема любых форм и в любой дозе витамина D, что также соответствует основным критериям для включения его в программу неонатального скрининга. В целом, полученные нами данные указывают на недооцененность частоты дефицита 24-гидроксилазы на территории нашей страны и заставляют задуматься о данной нозологии как кандидатной для расширения программы неонатального скрининга в РФ.

Учитывая повышенный риск развития гиперкальциурии и нефрокальциноза у гетерозиготных носителей патогенных вариантов в гене CYP24A1, считаем необходимым внедрение биохимического контроля (уровень кальция в крови и кальция в моче) при назначении холекальциферола в любых дозах и во всех возрастных группах.

## ДОПОЛНИТЕЛЬНАЯ ИНФОРМАЦИЯ

Источники финансирования. Работа выполнена по инициативе авторов без привлечения финансирования.

Конфликт интересов. Авторы декларируют отсутствие явных и потенциальных конфликтов интересов, связанных с содержанием настоящей статьи.

Участие авторов. Все авторы одобрили финальную версию статьи перед публикацией, выразили согласие нести ответственность за все аспекты работы, подразумевающую надлежащее изучение и решение вопросов, связанных с точностью или добросовестностью любой части работы.

Согласие пациента. Добровольные информированные согласия пациентов и их законных представителей на публикацию в журнале «Проблемы эндокринологии» получены.

Благодарности. Авторы выражают благодарность в предоставлении дополнительных материалов при подготовке данной статьи Д.Н. Хмельковой и А.А. Исаеву.
